# Hypoglycemia and Ketosis in the Setting of Pediatric Ethanol Intoxication: A Case Report

**DOI:** 10.7759/cureus.24538

**Published:** 2022-04-27

**Authors:** Jonathon Compton, John Azat Masoud, Edwin McCray, Anmol Singh, Harshit Terala, Fasil Mohomed

**Affiliations:** 1 Internal Medicine, Campbell University School of Osteopathic Medicine, Lillington, USA; 2 Emergency Medicine, Cape Fear Valley Medical Center, Fayetteville, USA; 3 Medicine, Firelands Regional Medical Center, Sandusky, USA; 4 Pediatrics, Southeastern Regional Medical Center, Lumberton, USA

**Keywords:** ketosis, ethanol metabolism, pediatric ethanol intoxication, ethanol intoxication, hypoglycemia

## Abstract

Acute alcohol intoxication is a common presentation to emergency departments. The intoxication of young pediatric patients is extremely rare. We present a case of a two-year-old female who presented to the emergency department with altered mentation, incontinence, and hypoglycemia. Over the next two days, the patient was treated with fluid replacement, and her hypoglycemia was managed. She was then discharged with no complications. While infant ethanol intoxication has a relatively low mortality rate, it can still be potentially fatal due to complications. Few studies have examined the morbidity associated with these presentations. This case demonstrates the importance of prompt identification and management to avoid a catastrophic outcome. Further research on the enzyme kinetics and long-term effects of early ethanol exposure resulting in hypoglycemia and ketosis is warranted.

## Introduction

Pediatric ethanol intoxication is rarely reported in the scientific literature. However, it is an important cause of poisoning in children. In 2012, there were 1,349 documented ethanol exposures to children under the age of five years, and in 2019, the Centers for Disease Control reported a single fatal ethanol exposure in a child under the age of five years [1,2]. Compared to adult livers, pediatric livers have 10-times less alcohol dehydrogenase (ADH), an enzyme integral to the hepatic metabolism of ethanol. Therefore, pediatric alcohol ingestion can lead to severe metabolic derangements [3]. Hypoglycemia is often seen in infant ethanol metabolism due to inhibition of gluconeogenesis and decreased glycogen storage compared to adults [4]. Although ethanol exposure is rarely fatal in the pediatric population, it is important to recognize alcohol intoxication as a potential diagnosis in children with symptoms of altered mental status, profound hypoglycemia, obtundation, and neurologic findings. This can be achieved by collecting blood alcohol levels and a urine drug screen on all pediatric patients presenting with altered mental status. We report a case of severe ethanol intoxication with hypoglycemia and ketosis in a two-year-old female. 

## Case presentation

A two-year-old female with a past medical history of premature birth, low birth weight, and fetal malnutrition presented to the emergency department (ED) with altered mental status per the mother. The gestational age of the mother was not provided. The child’s last known normal was bedtime at 12 AM, approximately eight hours before presentation. The mother reported that she attempted to wake the child in the morning and found that the child was lethargic, limp, and had urinated in the bed. Bed-wetting was a rare occurrence per the mother, and there was no known history of developmental or neurologic delays. The mother also reported no mental or physical changes noted when she put the patient to bed the night before and denied any known overnight events. The mother reports that she and her cousin were drinking “gin-and-juice” that night from the child’s sip-cups. The drinks were reported to contain a high concentration of alcohol relative to the juice.

**Figure 1:**
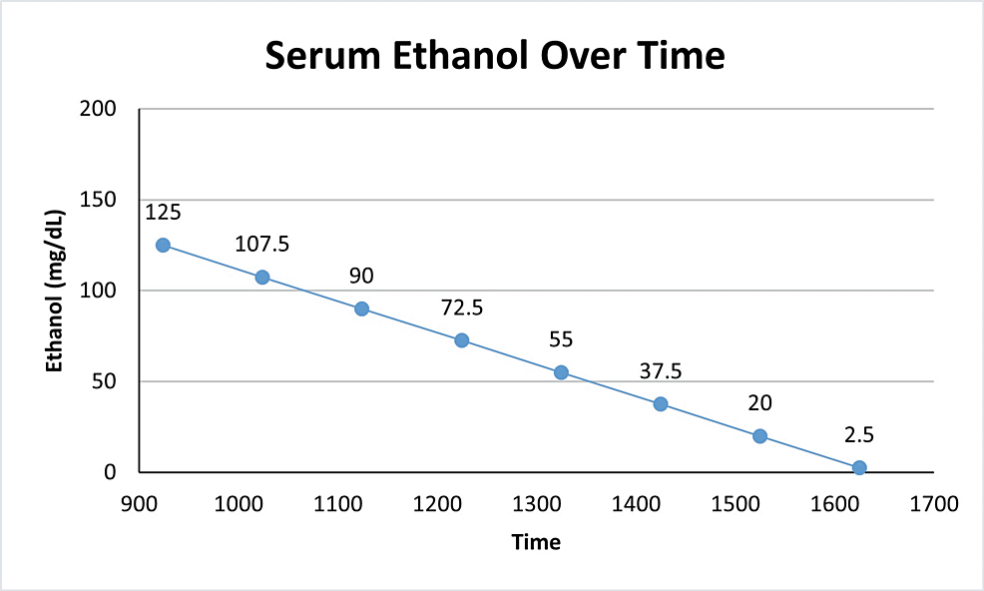
The presence of serum ethanol in our patient over the duration of her stay

Upon arrival at the ED, point of care glucose was less than 40 mg/dL. The patient’s most recent meal was reportedly chicken fingers and fries at 10:00 PM the night before. Upon presentation, the patient’s Glasgow coma score (GCS) was initially 6. The patient was also having abnormal, full-body twitching every 30 seconds. The patient was maintaining her airway, and intubation was not necessary. The patient received half an ampule of 50% dextrose, which raised blood glucose to 301 mg/dl and GCS to 14, due to a lack of spontaneous eye-opening. Arterial blood gas and comprehensive metabolic panel revealed a metabolic acidosis including a pH at 7.13, pCO2 37 mmHg, HCO3 12.3 mEq/L, Na 138 mEq/L, Cl 104 mEq/L, arterial lactate of 7.6 mmol/L, and an anion gap of 22. Serum ethanol was found to be 125 mg/dL. Beta-hydroxybutyrate was elevated to 18.3 mmol/L and procalcitonin to 2.45 ng/mL. Lab value trends are noted in Table 1 and Figure 1. Urinalysis revealed 3+ ketones. Her urine drug screen was negative and serum acetaminophen and salicylate screens were both within normal limits. Arterial blood gas (ABG) findings were consistent with metabolic acidosis with ketosis without evidence of respiratory acidosis. Chest X-ray, CT of the head, and electrocardiogram (ECG) were all unremarkable. Blood cultures were drawn but were ultimately negative. No episodes of nausea or vomiting were observed.

**Table 1:** Relevant lab values of the patient for the duration of her stay

	Time	Value	Hospital Day
Ethanol (mg/dL)			
	924	125	1
	1400	25	1
	1923	10	1
	2356	10	1
Beta-hydroxybutyrate (mg/dL)			
	1119	18.3	1
	1400	12.3	1
	1923	19.4	1
	243	14.3	2
	1402	0.8	2
Glucose (mg/dL)			
	859	<40	1
	912	363	1
	1119	93	1
Lactic Acid (mmol/L)			
	924	6.8	1
	1119	4.9	1
		4.4	2

The patient was admitted for further observation and management. Maintenance fluids were switched to 10% dextrose in 0.45% normal saline with bicarbonate (25 mEq) at 75 mL/hr to a target glucose infusion rate of 7 ml to 8 ml/hr. The patient was placed on a nil per oral (NPO) diet and monitored on telemetry. A routine electroencephalogram (EEG) was ordered due to the abnormal twitching. The EEG was read to be normal. Over the next two days, the patient improved to a GCS of 15 and lab values began to normalize. The patient was more alert and responsive, and her somnolence from the ethanol ingestion was also resolved. The patient was discharged three days after the initial presentation with no obvious residual effects.

## Discussion

Accidental alcohol intoxication among infants is rare in the United States, with a low mortality rate. Few studies are addressing the incidence and morbidity. The 2019 Annual Report of the American Association of Poison Control Centers (AAPCC) National Poison Data System showed 24 fatalities in children under five years old due to single substance exposure; of those cases, one fatality was attributed to ethanol ingestion, comprising 4.17% of single substance exposures under five years old [2].

One case of documented infant alcohol poisoning involved a nine-week-old male who ingested vodka provided by his grandmother in his formula resulting in a presenting blood alcohol level of 330 mg/dL and blood glucose of 167 mg/dL without acid-base disturbance [5]. Many published cases involve liquor inadvertently being used to dilute the baby formula. A unique aspect of this case report is the downtime during which the patient was alone and unobserved. The mother reported the child was at baseline when put to bed at 10:00 PM, which was thought to be after the ethanol exposure. The patient was then noted to have altered mentation at 8:00 AM the following morning, representing roughly 10 hours of uncertain signs, symptoms, or other potentially observable abnormalities. In adults, serum ethanol concentrations of 50 to 100 mg/dL can lead to acidosis, central nervous system disturbances, respiratory distress, hypoglycemia, and coma. Concentrations generally peak 30 to 60 minutes after exposure [6]. As our patient presented over eight hours after the predicted exposure, the peak ethanol concentration most likely was significantly higher than the 125 mg/dL found on the initial point of care glucose. 

Unfortunately, there are very few published studies on the enzyme kinetics in ethanol metabolism in patients of this age. It is thought that ADH plays a much smaller role in metabolism during childhood, and ethanol is perhaps metabolized by catalase and to a lesser extent, the cytochrome P450 pathways. Alcohol dehydrogenase works by converting ethanol into acetaldehyde, which is then converted into acetate. Acetate enters the bloodstream and is ultimately filtered and excreted renally. As mentioned above, this mechanism (as seen in Figure 1) is postulated to have a smaller role during childhood. Catalase is primarily located in the peroxisomes of cells and uses hydrogen peroxide to oxidize ethanol into aldehydes [7]. The average elimination rate in a non-tolerant adult is 15 to 20 mg/dL/hr [8]. 

Serum alcohol concentration appears to be determined by simple diffusion. A large amount of alcohol ingested creates a higher concentration gradient, leading to greater absorption. It also appears that time plays an important role as well. An amount of alcohol ingested over a short period will lead to a greater concentration than an equal amount ingested over a longer time. Alcohol dehydrogenase isoforms in the stomach are responsible for the first-pass metabolism of alcohol, limiting toxicity and absorption. Ingestion of alcohol during a fasting state promotes rapid gastric emptying into the duodenum and limits first-pass metabolism. At high serum concentrations, alcohol is metabolized by ADH at a constant rate, following zero-order kinetics. This is due to ADH’s relatively low Michaelis constant (Km), indicating a high affinity for ethanol. However, at lower concentrations, ethanol metabolism follows first-order kinetics since ADH does not reach saturation [9,10]. As mentioned previously, the developing liver has lower concentrations of ADH and is thought to be a slow metabolizer of ethanol.

Correction of pediatric hypoglycemia must also be carefully considered. The Pediatric Advanced Life Support (PALS) program recommends using dextrose 10 % in water (D10W) in the child patient. There are multiple risk factors in the use of dextrose 50 % in water (D50W) in the treatment of pediatric hypoglycemia. They include small vessel sclerosis, local tissue damage in case of extravasation, and thrombophlebitis [10]. Dextrose 50 % in water can also lead to hyperglycemia that later needs management with insulin and rebound hypoglycemia due to the significant release of insulin in response to the large amounts of glucose [10]. 

Limitations

We cannot make any generalizations about similar patients as this solely represents the clinical account of a single patient. Additionally, no causal relationships, statistically significant statistics, or external validity is present due to the nature of the study design.

## Conclusions

Ethanol ingestion in a young alcohol-naive patient may result in hypoglycemia, and ketosis is a potential finding. Hypoglycemia in a young alcohol consumer may be more common, requiring further research and delineation of the subject.

Our case highlights the importance of keeping a broad differential diagnosis and using diagnostic algorithms appropriately to provide quality patient care. With infant alcohol ingestion being a rare occurrence, both the presentation and morbidity are not well studied. In the face of clinical suspicion of pediatric ethanol intoxication, rapid identification and prompt treatment should be an urgent priority due to the potential for poor long-term health outcomes. It is important to identify hypoglycemia and ketosis in an unresponsive patient as a potential sign of child neglect. Further research on the enzyme kinetics and long-term effects of early ethanol exposure is warranted to develop evidence-based diagnostic and treatment protocols. In addition, further research could explore whether the differences in enzyme composition and metabolism of ethanol in children are different from adults due to being alcohol-naive. 
